# CYP2D6 Genetic Variation and Antipsychotic-Induced Weight Gain: A Systematic Review and Meta-Analysis

**DOI:** 10.3389/fpsyg.2021.768748

**Published:** 2022-02-03

**Authors:** Yanisa Wannasuphoprasit, Stig Ejdrup Andersen, Maria J. Arranz, Rosa Catalan, Gesche Jurgens, Sanne Maartje Kloosterboer, Henrik Berg Rasmussen, Anjali Bhat, Haritz Irizar, Dora Koller, Renato Polimanti, Baihan Wang, Eirini Zartaloudi, Isabelle Austin-Zimmerman, Elvira Bramon

**Affiliations:** ^1^Division of Psychiatry, University College London, London, United Kingdom; ^2^Clinical Pharmacological Unit, Zealand University Hospital, Roskilde, Denmark; ^3^Fundació Docència I Recerca, Mútua Terrassa, Barcelona, Spain; ^4^Barcelona Clinic Schizophrenia Unit, Hospital Clínic Barcelona, Institut d’Investigacions Biomèdiques August Pi i Sunyer, University of Barcelona, Barcelona, Spain; ^5^CIBERSAM, Centro de Investigación Biomédica en Red de Salud Mental, Madrid, Spain; ^6^Department of Hospital Pharmacy and Child and Adolescent Psychiatry, Erasmus MC, University Medical Center Rotterdam, Rotterdam, Netherlands; ^7^Institute of Biological Psychiatry, Mental Health Centre Sct Hans, Roskilde, Denmark; ^8^Department of Science and Environment, Roskilde University Center, Roskilde, Denmark; ^9^Division of Human Genetics, Department of Psychiatry, Yale School of Medicine, New Haven, CT, United States; ^10^Veterans Affairs Connecticut Healthcare System, West Haven, CT, United States; ^11^Institute of Psychiatry, Psychology and Neuroscience, King’s College London, London, United Kingdom; ^12^Institute of Cognitive Neuroscience, University College London, London, United Kingdom; ^13^Camden and Islington NHS Foundation Trust, London, United Kingdom

**Keywords:** CYP2D6, antipsychotic-induced weight gain, antipsychotic, weight gain, pharmacogenetic, personalized medicine, mental health

## Abstract

**Background:**

Antipsychotic-induced weight gain is a contributing factor in the reduced life expectancy reported amongst people with psychotic disorders. CYP2D6 is a liver enzyme involved in the metabolism of many commonly used antipsychotic medications. We investigated if *CYP2D6* genetic variation influenced weight or BMI among people taking antipsychotic treatment.

**Methods:**

We conducted a systematic review and a random effects meta-analysis of publications in Pubmed, Embase, PsychInfo, and CENTRAAL that had BMI and/or weight measurements of patients on long-term antipsychotics by their CYP2D6-defined metabolic groups (poor, intermediate, normal/extensive, and ultra-rapid metabolizers, UMs).

**Results:**

Twelve studies were included in the systematic review. All cohort studies suggested that the presence of reduced-function or non-functional alleles for *CYP2D6* was associated with greater antipsychotic-induced weight gain, whereas most cross-sectional studies did not find any significant associations. Seventeen studies were included in the meta-analysis with clinical data of 2,041 patients, including 93 poor metabolizers (PMs), 633 intermediate metabolizers (IMs), 1,272 normal metabolizers (NMs), and 30 UMs. Overall, we did not find associations in any of the comparisons made. The estimated pooled standardized differences for the following comparisons were (i) PM versus NM; weight = –0.07 (95%CI: –0.49 to 0.35, *p* = 0.74), BMI = 0.40 (95%CI: –0.19 to 0.99, *p* = 0.19). (ii) IM versus NM; weight = 0.09 (95% CI: –0.04 to 0.22, *p* = 0.16) and BMI = 0.09 (95% CI: –0.24 to 0.41, *p* = 0.60). (iii) UM versus EM; weight = 0.01 (95% CI: –0.37 to 0.40, *p* = 0.94) and BMI = –0.08 (95%CI: –0.57 to 0.42, *p* = 0.77).

**Conclusion:**

Our systematic review of cohort studies suggested that CYP2D6 poor metabolizers have higher BMI than normal metabolizers, but the data of cross-sectional studies and the meta-analysis did not show this association. Although our review and meta-analysis constitutes one of the largest studies with comprehensively genotyped samples, the literature is still limited by small numbers of participants with genetic variants resulting in poor or UMs status. We need further studies with larger numbers of extreme metabolizers to establish its clinical utility in antipsychotic treatment. *CYP2D6* is a key gene for personalized prescribing in mental health.

## Introduction

Antipsychotic drugs are an important treatment option for psychotic disorders including schizophrenia, bipolar disorder, and other psychoses (Taylor et al., 2021). Antipsychotics have been shown to improve psychotic symptoms with fewer relapses in the first year (Leucht et al., 2012) and reduce excess mortality in people living with schizophrenia (Taipale et al., 2018, 2020). However, their adverse effects, including extrapyramidal side effects in typical antipsychotics and metabolic side effects in atypical antipsychotics, can have an impact on physical health and quality of life and contribute to drug discontinuation (Lieberman et al., 2005; Leucht et al., 2013).

Antipsychotic-induced weight gain is a frequently reported adverse drug reaction, particularly to second-generation antipsychotics (Miyamoto et al., 2012), leading to increased risk for cardiovascular diseases, diabetes and some types of cancer (Newcomer, 2005; Osborn et al., 2013). These are some of the key reasons for the 10–20-year average reduction in life expectancy observed in people with schizophrenia (Chesney et al., 2014; Galderisi et al., 2021). The mechanisms of antipsychotic-induced weight gain include genetic, biological and psychosocial factors that are not fully understood. Indeed, the propensity to develop weight gain varies between individuals and different drugs and is dose-related (Müller and Kennedy, 2006; Simon et al., 2009; Tek et al., 2016). One approach to understand this inter-individual variability is through pharmacogenetic studies investigating how genetic variation can influence treatment response and tolerability.

The Cytochrome P450 family of liver enzymes include, among others, CYP1A2, CYP2D6, CYP2C19, and CYP3A4, which are essential for the metabolism of many commonly prescribed medications (Guengerich, 2008). CYP2D6 is involved with the metabolic pathway of approximately 40% of antipsychotic drugs, including aripiprazole, risperidone, haloperidol, chlorpromazine (Fleeman et al., 2011; Cacabelos et al., 2013). *CYP2D6* is a highly polymorphic gene with more than 100 known functional variants including null or increased function alleles with deletions or duplications respectively, as well as other variants resulting in reduced enzyme activity (Sim and Ingelman-Sundberg, 2013; Nofziger et al., 2020). Based on the combination of these alleles, participants can be categorized into four CYP2D6 phenotypic groups: poor metabolizers (PMs), intermediate metabolizers (IMs), normal metabolizers, (NMs), and ultra-rapid metabolizers (UMs) (Caudle et al., 2020). Therefore, PMs and IMs have higher drug plasma concentrations and may be more likely to experience adverse drug reactions, while UMs show lower drug plasma concentrations and potentially poorer efficacy (Ingelman-Sundberg, 2005; Nofziger et al., 2020).

*CYP2D6* genetic variation is shown to impact antipsychotic plasma concentrations in previous pharmacokinetic studies of aripiprazole, haloperidol and risperidone, where antipsychotic dosage and duration of treatment were controlled for (Van Der Weide and Van Der Weide, 2015; Sychev et al., 2017; Belmonte et al., 2018). Therefore, the varying plasma concentrations could influence the likelihood of patients developing adverse effects, including weight gain (Kloosterboer et al., 2021), and consequently, *CYP2D6* gene testing could contribute to inform appropriate antipsychotic prescribing in clinical settings (Taylor et al., 2021). In fact, *CYP2D6* genetic testing for several antipsychotics, including haloperidol, aripiprazole and risperidone, has already been recommended in clinical guidelines by the Dutch Pharmacogenetics Working Group (DPWG) (Swen et al., 2011).

Previous systematic reviews and meta-analyses investigating the putative relationship between CYP2D6 metabolic status and antipsychotic adverse effects highlighted that there is limited primary research, yielding inconclusive results (Lett et al., 2012; Maruf et al., 2020). The relationship between *CYP2D6* genotype and antipsychotic related weight gain has not been examined in a meta-analysis, despite being one of the most common adverse reactions to antipsychotics. Therefore, we here aim to conduct the first systematic review and meta-analysis to investigate whether *CYP2D6* genetic variation influences weight gain in people taking antipsychotic treatment.

## Materials and Methods

### Literature Search

We searched in Pubmed, Embase, PsychInfo, and CENTRAL databases for original peer-reviewed papers published in English from January 1995 to April 2021 (both inclusive), using the following search terms: ‘(Cytochrome* or CYP* or P450 or CYP2D6) AND (antipsychotic* or neuroleptic* or risperidone or olanzapine or thioridazine or perphenazine or fluphenazine or zuclopenthixol or haloperidol or chlorpromazine or clozapine or quetiapine or ziprasidone or benperidol or methotrimeprazine or pimozide or sulpiride or trifluoperazine or amisulpride or sertindole or zotepine or aripiprazole) AND (genot* or allel* or pharmacogenetic* or pharmacokinetic*) AND (BMI or weight or obes* OR body mass index or ADRs or ADR or adverse drug reactions)’. Of note, this list of medications was chosen from Drug Bank (Wishart et al., 2006), the Maudsley Prescribing guideline (Taylor et al., 2021) and review papers (Baptista et al., 2005; Fleeman et al., 2011; Leucht et al., 2013) as medications that could be metabolized via CYP2D6 pathway or have weight gaining effects. Additionally, we performed a Google Scholar search for review articles that looked at pharmacogenetics of antipsychotics, focusing on *CYP2D6* polymorphisms and metabolic side effects and examined their reference lists for additional relevant articles.

### Inclusion Criteria and Data Extraction

The inclusion criteria were: (1) randomized control trials or observational studies conducted in patients taking antipsychotic drugs; (2) genotyped one or more *CYP2D6* alleles; and (3) BMI and/or weight of the participants were recorded. The studies had to meet all three inclusion criteria to be considered eligible to be included in the systematic review and/or meta-analysis.

The titles and abstracts were initially assessed by two independent reviewers. Full text articles were assessed for eligibility, and the authors of eligible records were contacted for unpublished data when necessary.

Some authors published open-source data with weight and BMI by CYP2D6 metabolic groups. Several studies published weight and BMI by *CYP2D6* allelic variants, while others provided us their database with all the *CYP2D6* allelic variants of each participant. In the latter, we classified participants into CYP2D6 metabolic groups from their star alleles using the consensus method, which was standardized for consistency between the Clinical Pharmacogenetics Implementation Consortium (CPIC) and Dutch Pharmacogenetics Working Group (DPWG) (Caudle et al., 2020). Each star allele is assigned an activity score: the activity score for a non-functional allele (e.g., *3, *4 and *5; gene deletion) is 0, for a reduced function allele (e.g., *41) is 0.5 (except that of *10 which is 0.25), for a normal function allele (e.g., *1 and *2) is 1, and for an increased function allele (e.g., *1xN and *2xN; gene duplication) is more than 1 (Supplementary Table 1). The activity score of each of the star alleles is then summed to give a total activity score for an individual. Lastly, the individual is assigned into CYP2D6 metabolic groups according to their total activity score (Supplementary Table 2).

### Statistical Analysis

We performed a meta-analysis with the Review Manager 5.2 software (The Cochrone Collaboration, 2014), using an inverse variance-weighted random effects model. A random effects model was chosen due to the moderate heterogeneity between the included studies. The comparisons of standardized mean differences of weight (kg) and BMI (kg/m^2^) were made using Hedges’ adjusted model between the following groups (i) PMs versus NMs, (ii) IMs versus NMs, (iii) UMs versus NMs, and (iv) PMs and IMs combined versus NMs and UMs combined. Where more than one weight and/or BMI measurements per subject were available, the readings at the end of the follow-up period were included. Tau^2^, chi^2^, and I^2^ tests were performed to assess heterogeneity between studies.

Additionally, we performed the following sensitivity analyses: choice of statistical test (random or fixed effects model), age group (adult or children and adolescents), relevant diagnoses (schizophrenia or other diagnoses) and medications taken (risperidone monotherapy or other medications). We did the analyses on the comparisons with the highest number of included studies for weight and BMI.

To find evidence of publication bias, we plotted a Funnel plot and performed an Egger’s regression (Egger et al., 1997) in the analyses that included more than ten studies, using Jamovi software (Version 1.6), an interface based on R package Metafor (Viechtbauer, 2010; The Jamovi Project, 2021).

## Results

### Literature Search

**FIGURE 1:**
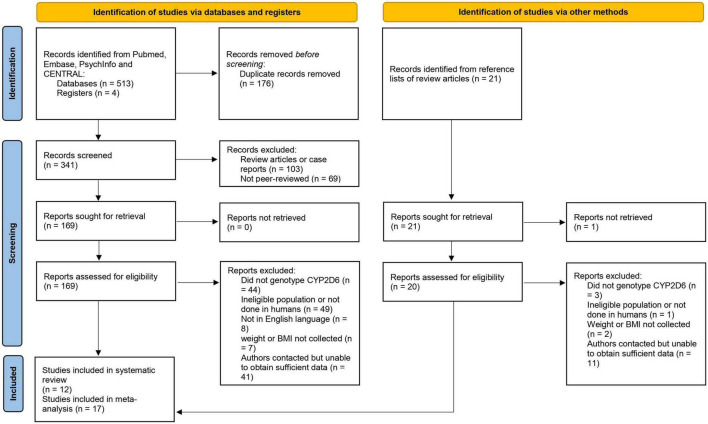
PRISMA flow diagram.

The literature search yielded 517 records. We excluded 174 duplicates, 103 review articles and 69 conference abstracts. Subsequently, 171 reports were assessed by two reviewers and 17 studies were included in the meta-analysis and 12 in the systematic review (see Figure 1 for the PRISMA flow diagram. Template taken from Page et al., 2021). The reasons for excluding the records included insufficient information on weight or BMI (Inada et al., 2003; Plesnicar et al., 2006; Subuh Surja et al., 2008) and ineligible study population such as healthy volunteers receiving a single drug dose (Sun et al., 2019) or schizophrenic patients with acute episodes who were on antipsychotic medication for less than one month before weight measurement (Vandenberghe et al., 2015). Some had genotyped some *CYP2D6* single nucleotide polymorphisms (SNPs) but, unfortunately, these selected SNPs did not allow us to call for *CYP2D6* alleles and classify participants into CYP2D6 metabolic groups (Lee et al., 2012).

Seven studies were included in the systematic review, but not in the meta-analysis, because they only published the conclusion of the analysis onto their paper, but not the analysis itself. Meanwhile, 11 studies in the meta-analysis were not included in the systematic review as their primary research outcomes were not weight and/or BMI.

### Systematic Review Findings

Twelve studies were included in the systematic review, six of which were cohort observational studies (Table 1), while the others were cross-sectional observational studies (Table 2). All six cohort studies showed evidence supporting the influence of *CYP2D6* genetic variation on antipsychotic-induced weight gain.

**TABLE 1:** The characteristics of the cohort observational studies included in the systematic review.

*Study*	*N*	*Ethnicity*	*Mean age* ± *SD (range) in years*	*Gender, % Male*	*Antipsychotics*	*Mean dose* ± *SD (range)*	*Duration of treatment*	*Diagnosis*	*CYP2D6 alleles genotyped*
Lu et al., 2021	76	Not reported (China)	45 (35–54)	100%	Risperidone	5 (2–6) mg/day	8 weeks	Schizophrenia	*2, *10, and *65
Jallaq et al., 2021	277	European descents (83.3%), African–Caribbean (10.8%)	14.3 ± 2.4 (6.0–19.6)	34.3%	Aripiprazole	Maximum dose 30 mg/day	Not reported	Mood disorder (e.g. bipolar, major depressive disorder)	*2A, *3, *4, *5, *6, *7, *8, *9, *10, *11, *14, *15, *17, *18, *19, *20, *40, *41, *42, and *44.
Nussbaum et al., 2014	81	Not reported (Romania)	15.7 (9–20)	46%	Risperidone, Aripiprazole, Olanzapine	Not reported	At least 18 months	Schizophrenia, bipolar disorders	*4
Correia et al., 2010	45	European descents (97.8%)	8.7 ± 4.30 (3–21)	75.6%	Risperidone	1.3 ± 0.7 (0.5–3) mg/day	At least 12 months	Autism	*3, *4, *5, *6 and gene duplication.
Lane et al., 2006	123	Asian (Hans Chinese)	34.0 ± 9.7	55.3%	Risperidone	4.0 ± 1.4 mg/day (at end point – day 42)	42 days	Schizophrenia with acute exacerbations	*10 (188-C/T)
Ellingrod et al., 2002	11	European descents	35.6 ± 5.5	100%	Olanzapine	14.3 ± 3.4 mg/day	Mean 13.8± 13.6 months	Schizophrenia	*3 and *4

**TABLE 2:** The characteristics of the cross-sectional observational studies included in the systematic review.

*Study*	*N*	*Ethnicity*	*Mean age* ± *SD (range) in years*	*Gender, % Male*	*Antipsychotics*	*Mean dose* ± *SD (range)*	*Duration of treatment*	*Diagnosis*	*CYP2D6 alleles genotyped*
Sukasem et al., 2018	89	Not reported (Thailand)	10.0 (8.9–13.4)	91.0%	Risperidone	1 (0.5–1.5) mg/day	63.9 (40.4–83.5) months	Autism-spectrum disorder	*4, *10, *5, *41 and gene duplication.
Dos Santos-Júnior et al., 2016	120	Not reported (Brazil)	13 ± 3.1 (8–20)	81.7%	Risperidone	0.04 ± 0.03 mg/kg/day	Not reported	Mental and behavioral disorders	*10 (c.100C > T)
Dodgen et al., 2015	24	European descents (58.3%), African-Caribbean (41.7%)	32.9 ± 12.4 (18–61)	66.7%	Risperidone	3.9 ± 1.8 mg/day	Not reported	Any psychiatric disorders	using an array with multiple SNPs for *CYP2D6* (at least 10 alleles genotyped)
Suzuki et al., 2014	66	Not reported (Japan)	37.4 ± 15.0	51.5%	Risperidone	4.8 ± 2.5 mg/day	At least 4 weeks	Schizophrenia	*5 and *10
Vanwong et al., 2014	86	Not reported (Thailand)	9.4 ± 3.6	83.7%	Risperidone	0.9 ± 0.8 mg/day	At least 2 weeks, 37.8 ± 20.3 months	Autism	*4, *5, *10, and *11
Mihara et al., 2000	101	Not reported (Japan)	48 ± 11	30.7%	Haloperidol	12 mg/day (all subjects)	At least 2 weeks	Schizophrenia	*5 and *10

Among the cohort studies identified, Jallaq et al. (2021) reported CYP2D6 phenotypes to be significantly associated with BMI percentile change (*p* = 0.038) in children and adolescents taking aripiprazole, although the direction of the effect was unclear from the box plot available. Other significant factors seen to influence BMI change included duration of aripiprazole treatment and the number of CYP2D6 substrate medications taken by each subject. Another study (Correia et al., 2010) in children and adolescents taking risperidone found significantly lower increase in BMI of 4.8% and in waist circumference of 5.8% in UMs compared to NMs, supporting a link between CYP2D6 metabolic phenotypes and antipsychotic-induced weight gain. However, this was contradicted by the findings in PMs whose increase in waist circumference appeared lower compared to NMs by 4%, possibly due to the small number of PM subjects.

The rest of the cohort studies looked at specific *CYP2D6* alleles, rather than *CYP2D6* metabolic phenotypes. Ellingrod et al. (2002) identified greater BMI percentage change in adult participants taking olanzapine with CYP2D6*3 or CYP2D6*4 alleles (more than 125%) compared to those without the non-functional alleles (just below 115%; *p* = 0.0097), although this study was limited by a small sample size of 11 participants. Supporting this study was a study by Nussbaum et al. (2014) who followed children and adolescents on risperidone, aripiprazole and olanzapine for 18 months, and identified significantly increased BMI in those with CYP2D6*4 alleles from 6 months follow-up onward. By the end of the 18-month follow-up, subjects with one non-functional allele had a mean BMI increase of 8.12 kg/m^2^, which was significantly greater than that of subjects with two wild-type copies (2.27 kg/m^2^; *p* < 0.001). Additionally, Lane et al. (2006) found evidence that adult patients receiving risperidone with CYP2D6 *1/*10 and CYP2D6 *10/*10 genotypes had a significantly higher weight of 1.138 kg and 0.799 kg respectively at the end of 42-day follow-up period, compared to CYP2D6 *1/*1 carriers. This was confirmed by Lu et al. (2021) who also investigated CYP2D6*10 and suggested that this variant was associated with weight gain and increased BMI compared to CYP2D6*2 and CYP2D6*65 carriers. Reports from the same study also suggested that those with the *C2851T* allele (the defining allele for CYP2D6*2) had less weight gain. However, it appeared that the overall trend for all participants from Lu et al.’s (2021) study ended up with lower weight and BMI after four and eight weeks of initiating risperidone, which contradicted previous literature that supported the weight gaining effect of risperidone (Muench and Hamer, 2010; Leucht et al., 2013; Avrahami et al., 2021).

On the contrary to the findings from cohort observational studies, only one out of six cross-sectional observational studies found a significant association between CYP2D6 metabolic phenotypes and antipsychotic-induced weight gain (Table 2). Dos Santos-Júnior et al. (2016) identified a significant association between the number of CYP2D6*10 alleles and occurrence of obesity in children and adolescents receiving risperidone. All cross-sectional studies involved risperidone in either adults or children and adolescents except one (Mihara et al., 2000). Of note, Vanwong et al. (2014) and Sukasem et al. (2018) could have some partial sample overlap.

### Meta-Analysis Findings

Seventeen studies were included in the meta-analysis and their characteristics are summarized in Table 3. In total, we identified 83 PMs, 566 IMs, 1137 extensive metabolizers and 30 UMs with weight measurements available and 71 poor, 451 intermediate, 875 normal, and 21 UMs with BMI measurements available.

**TABLE 3:** Characteristics of the studies included in the meta-analysis.

Study	*N*	Ethnicity	Mean age ± *SD (range) in years*	Gender, %Males	*CYP2D6* Genotyping	Diagnosis	Antipsychotic drugs	Outcomes	Summary of findings
Jürgens et al., 2020	290	Not reported (Denmark)	41.4 (30–53)	54.3%	*3, *4, *5, *6 and gene duplication.	Schizophrenic spectrum	Various antipsychotics, e.g., aripiprazole, risperidone, and clozapine. Dose and treatment duration at baseline not reported.	BMI and weight	N/A
Jallaq et al., 2021	277	European descents (83.3%), African-Caribbean (10.8%)	14.3 ± 12.5 (6.0–19.6)	34.3%	*2A, *3, *4, *5, *6, *7, *8, *9, *10, *11, *14, *15, *17, *18,*19, *20, *40, *41, *42, and *44.	Mood disorders (bipolar mania, major depressive disorder or disruptive mood dysregulation disorder)	Aripiprazole. 56.7% were on 5 mg or more of Aripiprazole. Duration of treatment 367.8 ± 464.7 days	Weight	BMI percentage change was associated with CYP2D6 phenotype groups.
Kloosterboer et al., 2021	40	European descents (Dutch origin) (78.6%)	Median age 9.7 ± 5.3 (6–18).	76.2%	*3, *4, *5, and *41	Autism-spectrum disorder	Risperidone. Median dose = 1.0 ± 0.5 mg per day. Median duration of treatment = 5.7 ± 4.8 months	BMI and weight	N/A
Ortega-Vázquez et al., 2021	48	Not reported (Mexico)	38.65 ± 10.53	58.3%	rs28371706, rs1065852, rs3892097, rs35742686 and gene duplication	Schizophrenia, Schizoaffective disorder and bipolar disorder.	Clozapine. Mean dose 188.75 ± 141.83 (10-700) mg/day for at least 6 months.	BMI and weight	N/A
Arranz et al., 2019	163	Not reported (Spain)	47.6 ± 13.6	45.9%	*2, *3, *4, *5, *6, *9, *10, *35, *41 and gene duplication	Schizophrenia, Schizoaffective and delusional disorder.	Various antipsychotics, e.g., clozapine, risperidone, and olanzapine. Mean olanzapine dose-equivalent 11.0 ± 6.4 mg/day. Treatment duration at baseline not reported.	BMI and weight.	N/A
Kiss et al., 2019	93	Not reported (Hungary)	31 (18–65)	44.1%	*3, *4, *5, *6, *10, and *41	Schizophrenia, bipolar disorders	Aripiprazole. Mean dose 15 mg/d (5–30 mg/d) for at least 4 weeks.	Weight	N/A
Akamine et al., 2017	41	Not reported (Japan)	36.4 ± 12.5.	24.4%	*1, *2, *5, and *10	Schizophrenia	100–600 mg per day of Clozapine for at least 4 weeks	Weight	N/A
Sychev et al., 2017	54	European descents (Russian and Tatar)	43.6 ± 13.5	49.4%	*4	Schizophrenia	Haloperidol monotherapy. Mean dose 12.6 mg/day (SD 4.2 mg/day).	Weight	N/A
Ivanova et al., 2016	475	European descents (100%)	40 (17–80)	Not reported	*3 and *4	Schizophrenia	Multiple drugs: Haloperidol, Chloroprotixene, Chloropromazin, Trifluoperazin, Clopiksol, and Risperidone.	BMI and weight	N/A
Dos Santos-Júnior et al., 2016	120	Not reported (Brazil)	13.0 ± 3.1 (8–20)	81.7%	*10	Mental and behavioral disorders	Risperidone. Mean dose 21.1 mg/day (SD 1.3 mg/day). Mean duration of treatment 25.9 ± 27.2 months	BMI	The presence of *10 allele was associated with occurrence of obesity.
Nussbaum et al., 2014	81	Not reported (Romania)	15.7 (9–20)	46%	*4	Schizophrenia, Bipolar disorders	Either: Risperidone, Aripiprazole, or Olanzapine. Dose and treatment duration not reported.	BMI was recorded at 0, 3, 6, 12, and 18 months.	No significant results at 0–3 months. Those with one *4 allele had significantly higher BMI than those with no ^*^ alleles at 6–18 months.
Suzuki et al., 2014	66	Not reported (Japan)	37.4 ± 15.0	51.5%	*5 and *10	Schizophrenia	Risperidone. Mean dose 4.8 ± 2.5 mg/day for 4 weeks.	Weight	No statistically significant difference in weight between those with 2 mutant alleles, those with 1 mutant allele and those with wild type alleles.
Roke et al., 2013	46	European descents (98%)	14.7 ± 2.1 (10 to 19).	100%.	*3, *4 *5, *6 and gene duplication.	Autism spectrum disorder or Disruptive behavior disorder as well as any psychiatric disorder.	Risperidone. Mean dose 1.6 mg/day or 0.026 mg/kg. Mean duration of treatment 4.4 ± 2.4 years.	BMI and weight	N/A
Bigos et al., 2011	178	118 European descents and 60 African–Caribbean	Not reported	73%	*4	Schizophrenia	7.5–30 mg of Olanzapine per day. Treatment duration not reported.	BMI and weight	N/A
Mihara et al., 2003	85	Not reported (Japan)	44.6 ± 14.4.	31.8%	*2, *3, *4, *5, and *10	Schizophrenia	No medication for at least 2 weeks followed by 3 mg of Risperidone twice a day for at least 2 weeks.	Weight	N/A
Ellingrod et al., 2002	11	European descents	35.5 ± 5.4	100%	*3 and *4	Schizophrenia	Olanzapine. Mean dose 14.2 ± 3.3 (ranges: 7.5–20) mg/day. Mean duration of treatment 13.8 ± 12.9 months.	BMI	Those with *1/*3 or *1/*4 genotype had significant BMI increase compared to those with *1/*1.
Mihara et al., 2000	101	Not reported (Japan)	48 ± 11.	30.7%	*3, *4, *5, and *10	Schizophrenia	12 mg/day of Haloperidol for at least 2 weeks.	Weight	N/A

All the characteristics reported, including age, gender and antipsychotic medications, are of the whole study population of the papers. *N/A means the main outcomes of the studies were other side effects of antipsychotic medications rather than weight or BMI. The relationship between CYP2D6 genetic variation and weight or BMI was not published in these studies. Unpublished data was acquired from contacting the authors.

#### Meta-Analysis Comparing CYP2D6 Poor Metabolizers and Normal Metabolizers

**FIGURE 2:**
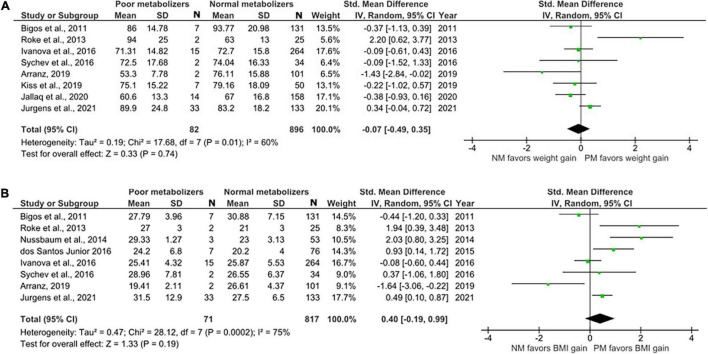
Forest plots comparing **(A)** standardized mean weight differences **(B)** standardized mean BMI differences between Poor metabolizers and Normal metabolizers. Random effects model and inverse variance method were use. CI, confidence interval; df, degree of freedom; N, number of participants; SD, standard deviation; SMD, standardized mean difference.

Ten studies were included in this comparison (Figure 2). Nussbaum et al. (2014) and Dos Santos-Júnior et al. (2016) only had the data for BMI available, while Kiss et al. (2019) and Jallaq et al. (2021) only had weight measurements. 93 PMs and 1025 NMs were identified in total. Weight and BMI were not significantly different between PMs and NMs with the standardized mean difference (SMD) for weight = –0.07 (95% CI: –0.49 to 0.35, *p* = 0.74) and SMD for BMI = 0.40 (95% CI: –0.19 to 0.99, *p* = 0.19). The results of the *I*^2^ test for both weight (60%) and BMI (75%) point at substantial heterogeneity between the primary studies.

#### Meta-Analysis Comparing CYP2D6 Intermediate Metabolizers and Normal Metabolizers

**FIGURE 3:**
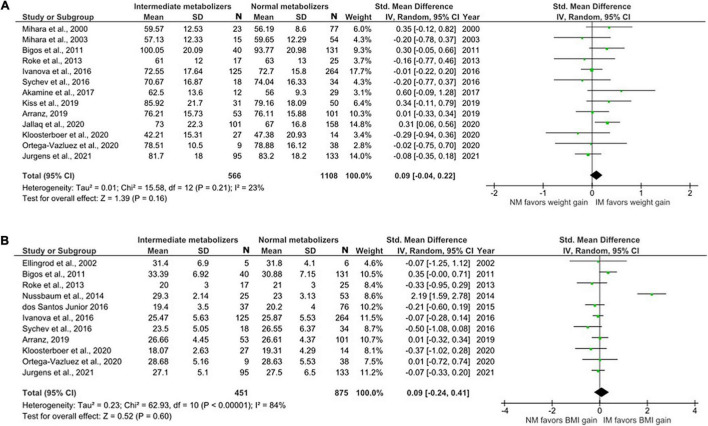
Forest plots comparing **(A)** standardized mean weight differences **(B)** standardized mean BMI differences between Intermediate metabolizers and Normal metabolizers. Random effects model and inverse variance method were use. CI, confidence interval; df, degree of freedom; N, number of participants; SD, standard deviation; SMD, standardized mean difference.

Sixteen studies with weight data for 566 IMs and 1108 NMs and BMI data for 451 IMs and 875 NMs were included in this comparison (Figure 3). The analysis showed no significant difference in weight and BMI between IMs and NMs (Weight: SMD = 0.09, 95%, CI: –0.04 to 0.22, *p* = 0.16; BMI: SMD = 0.09, 95% CI: –0.24 to 0.41, *p* = 0.60). The *I*^2^ of the heterogeneity test for weight was 23%, suggesting low to moderate heterogeneity, whereas that of BMI was 84%.

#### Meta-Analysis Comparing CYP2D6 Ultra-Rapid and Normal Metabolizers

**FIGURE 4:**
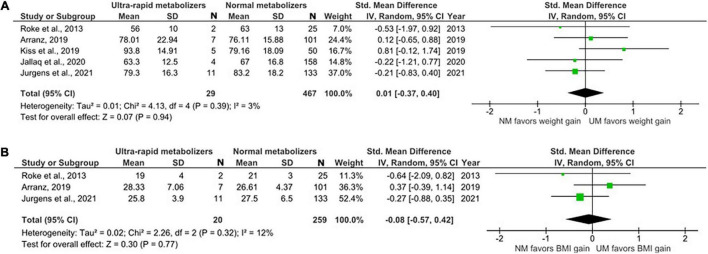
Forest plots comparing **(A)** standardized mean weight differences **(B)** standardized mean BMI differences between Ultra-rapid metabolizers and Normal metabolizers. Random effects model and inverse variance method were use. CI, confidence interval; df, degree of freedom; N, number of participants; SD, standard deviation; SMD, standardized mean difference.

The data for UMs were limited. We were able to include five studies with only a total of 505 NMs and 29 UMs (Figure 4). There was no evidence of weight or BMI differences in UMs compared to NMs (weight; SMD = 0.01, 95% CI: –0.37 to 0.40, *p* = 0.94 and BMI; SMD = –0.08, 95% CI: –0.57 to 0.42, *p* = 0.77).

#### Grouped Analyses, Sensitivity Analyses, and Tests for Publication Bias

**FIGURE 5:**
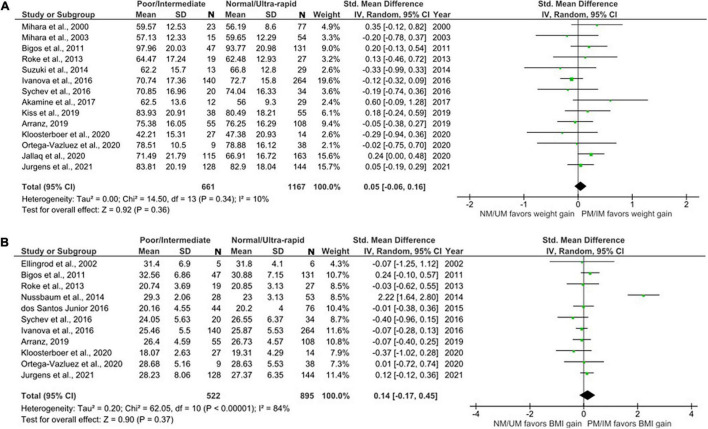
Forest plots comparing **(A)** standardized mean weight differences **(B)** standardized mean BMI differences between Poor metabolizers and Intermediate metabolizers combined compared to Normal metabolizers and Ultra-rapid metabolizers combined. Random effects model and inverse variance method were use. CI, confidence interval; df, degree of freedom; N, number of participants; SD, standard deviation; SMD, standardized mean difference.

To increase statistical power, we conducted a secondary analysis to compare between group 1: PMs and IMs combined (*N* = 661) and group 2: NMs and UMs combined (*N* = 1167). No statistically significant differences were found (Figure 5).

We also performed sensitivity analyses for these group analyses to evaluate the following variables; choice of statistical test (random or fixed effects model), age (adult or children and adolescents), diagnoses (schizophrenia or other diagnoses), and medications (risperidone monotherapy or other medications). None of the sensitivity analyses yielded significant results (Table 4). This was consistent with the main analyses and, therefore, supporting the robustness of the main analyses.

**TABLE 4:** Table summarizing results from sensitivity analyses for the comparison of standardized mean weight/BMI between (1) PM and IM combined versus (2) NM and UM combined.

Sensitivity analysis category	Weight						BMI					
	Studies	PM/IM	NM/UM	Standardized mean difference [95% CI]	*P*-value	I-squared	Studies	PM/IM	NM/UM	Standardized mean difference [95% CI]	*P*-value	I-squared
Original (random effects)	**14**	**661**	**1167**	**0.05 [–0.06, 0.16]**	**0.36**	**10%**	**11**	**522**	**895**	**0.14 [–0.17, 0.45]**	**0.37**	**84%**
Fixed effects	14	661	1167	0.05 [–0.05, 0.14]	0.34	10%	11	522	895	0.07 [–0.04, 0.19]	0.19	84%
Adult	11	500	963	0.02 [–0.10, 0.13]	0.77	5%	7	404	725	0.01 [–0.12, 0.13]	0.91	0%
Children and adolescents	3	161	204	0.15 [–0.10, 0.40]	0.25	13%	4	118	170	0.45 [–0.64, 1.54]	0.42	94%
Schizophrenia	9	453	870	0.01 [–0.12, 0.15]	0.86	19%	6	395	687	0.01 [–0.13, 0.14]	0.92	10%
Other diagnosis	5	208	297	0.16 [–0.02, 0.34]	0.08	0%	5	127	208	0.37 [–0.53, 1.26]	0.42	92%
Risperidone monotherapy	4	74	124	–0.16 [–0.47, 0.15]	0.31	0%	3	90	117	−0.08 [–0.37, 0.20]	0.57	0%
Other medications	10	587	1043	0.08 [–0.04, 0.20]	0.19	20%	8	432	778	0.24 [–0.15, 0.64]	0.23	88%

**FIGURE 6:**
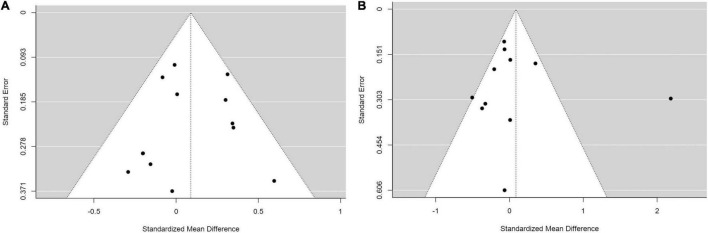
Funnel plots with studies comparing **(A)** standardized mean weight differences **(B)** standardized mean BMI differences between Intermediate metabolizers and Normal metabolizers.

**FIGURE 7:**
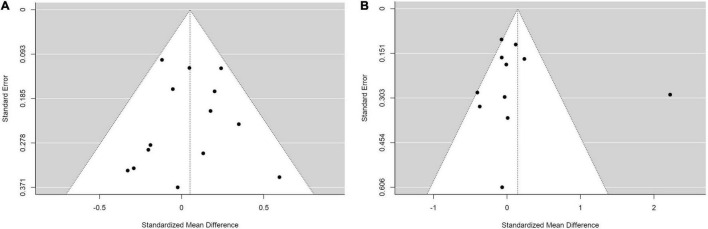
Funnel plots with studies comparing **(A)** standardized mean weight differences **(B)** standardized mean BMI differences between Poor metabolizers and Intermediate metabolizers combined compared to Normal metabolizers and Ultra-rapid metabolizers combined.

Finally, we assessed publication bias using a Funnel plot and an Egger’s regression analysis. We did the analysis in the comparisons that included more than ten studies, which were of comparisons of SMDs of (1) weight between IMs and NMs (coefficient = –0.310, *p* = 0.757; Figure 6A), (2) BMI between IMs and NMs (coefficient = 0.050, *p* = 0.960; Figure 6B), (3) weight between PMs and IMs combined versus NMs and UMs combined (coefficient = –0.155, *p* = 0.877; Figure 7A), and (4) BMI between PMs and IMs combined versus NMs and UMs combined (coefficient = 0.055, *p* = 0.956; Figure 7B). These Egger’s regression analysis did not show evidence of publication bias. The funnel plots for the comparisons with weight measurements were symmetrical indicating no evidence of publication bias, whereas those for BMI appeared asymmetrical with fewer studies on the significant results side, suggesting that there may be a possible bias or other factors influencing the symmetry. However, the asymmetry seen is on the opposite side to what one would expect if there is publication bias. It is worth noting that 11 out of the 17 included studies did not publish their weight or BMI data. This data was kindly shared with us upon request to the authors. Hence, inclusion of these primary papers may reduce the publication bias. We did not test publication bias for the rest of the group comparisons since there were only between three and eight primary studies thus limiting statistical power to detect publication bias.

## Discussion

To our knowledge, this study is the first comprehensive systematic review and meta-analysis of the literature on the relationship between *CYP2D6* genetic variation and antipsychotic-induced weight gain. Considering that weight gain is one of the most common adverse effects in second-generation antipsychotics and CYP2D6 enzyme plays a key role in the metabolism of many antipsychotics, there were surprisingly only five papers that specifically investigated this relationship. Weight gain can affect quality of life and more research on this topic will help us understand how genetic testing can be used to guide antipsychotic prescribing.

In our systematic review, all cohort studies found evidence that genetic variation resulting in decreasing CYP2D6 function was associated with antipsychotic-induced weight gain, and increased function alleles were associated with lower gain in weight or BMI. However, the systematic review of cross-sectional studies and our meta-analysis did not support this and found no significant differences between CYP2D6 metabolic groups upon weight or BMI. This inconsistency within the literature available could be due to the size and quality of primary studies and highlights the importance of careful study designs that account for the frequency of relevant genetic variants as well as other relevant variables that could influence weight gain.

Some of the cohort studies investigated specific *CYP2D6* allelic variants rather than CYP2D6 metabolic groups and found associations between specific *CYP2D6* alleles and antipsychotic-induced weight gain. This was mostly supported by previous literature since several sources (Gaedigk et al., 2008; Hicks et al., 2017; Nofziger et al., 2020) coincided to find enough pharmacokinetic evidence to conclude that CYP2D6*3 and CYP2D6*4 are non-functional alleles, whereas CYP2D6*10 is a reduced-function allele. These alleles result in increased plasma antipsychotic concentrations and may therefore increase the likelihood of developing antipsychotic-induced weight gain. Meanwhile, CYP2D6*2 is believed to result in normal metabolism, which contradicts the findings in Lu et al. (2021).

In this meta-analysis, we did not find evidence of a relationship between *CYP2D6* genetic variation and antipsychotic-induced weight gain. The comparison of weight and BMI for PMs included only 93 patients in this group and high heterogeneity between primary studies, which limited statistical power. The small sample size reflected the low prevalence of PMs in general population ranging from 1% in East Asians to 7% in individuals of European descent (Ozawa et al., 2004). Correspondingly, the number of UMs included in the analysis was even smaller and making the comparison most likely underpowered. Considering the comparisons for IMs with more than 400 participants in this group, power was not a concern, and our data showed no evidence of a change on antipsychotic-induced weight gain in IMs. Several factors could have confounded the analyses and we will discuss these in the following paragraphs.

Firstly, the demographic characteristics of the participants varied considerably between studies, including age groups, ethnicities, male to female ratio and the diagnoses, and this contributed to the high heterogeneity found between primary studies as indicated by the sensitivity analysis (Table 4). The group analysis with BMI measurements showed high heterogeneity with I-squared of 84% (Figure 5B). Further subgroup analyses have shown high heterogeneity in the following subgroups: the children and adolescents age group, those having diagnoses other than schizophrenia and those taking medications other than risperidone, whereas their counterpart subgroups did not show evidence of heterogeneity. These analyses provide evidence that these studies are the source of the significant heterogeneity between studies for analyses involving BMI measurements.

Previous research (Strassnig et al., 2007; Gebhardt et al., 2009) shows that female gender and younger age are risk factors for developing antipsychotic-induced weight gain. While African Americans appear to have increased risk of antipsychotic-induced weight gain compared to individuals of European descent, East Asians show reduced risk (Chan et al., 2013; Tek et al., 2016). When comparing weight gain in children and adolescents, it is also recommended to consider their baseline weight and height and methods like using a *z*-score should be considered (Martínez-Ortega et al., 2013). Unfortunately, we could not control for these variables in our meta-analysis and if more studies were available, meta-regressions accounting for ethnicity, age, sex, drugs, and other key variables should be conducted.

Secondly, some studies in the meta-analysis (Dodgen et al., 2015; Jallaq et al., 2021) performed extensive genotyping using *CYP2D6* arrays, whereas others (Bigos et al., 2011; Nussbaum et al., 2014; Dos Santos-Júnior et al., 2016) only genotyped one or very few single nucleotide polymorphisms. Although these are the most common non-functional and reduced function alleles in European and North American population, the studies in question may have missed other allelic variants that could influence CYP2D6 metabolic activity and mislabeled participants as NMs, leading to fewer numbers of poor, intermediate, or UMs identified (Owen et al., 2009; Gaedigk et al., 2017).

Thirdly, the hypothesis of this study was based on pharmacokinetic studies that found evidence supporting the effect of *CYP2D6* gene variation on plasma concentration of many, but not all, antipsychotic medications (Brockmöller et al., 2002; Kubo et al., 2007; Dahl, 2012). These pharmacokinetic findings may not necessarily translate to varying degrees or incidences of adverse effects (Simon et al., 2009). One reason is that antipsychotics can be metabolized using several metabolic pathways, involving many Cytochrome P450 enzymes (Urichuk et al., 2008). The main metabolic pathway for a particular drug may involve other CYP450 enzymes besides CYP2D6 and, furthermore, certain antipsychotics such as clozapine and olanzapine may inherently have more propensity to cause weight gain (Lett et al., 2012; Leucht et al., 2013). Therefore, antipsychotic choice can greatly influence the amount of weight gain measured. In addition to the drug choice, duration of treatment (Spina and De Leon, 2007; Tek et al., 2016). Jones et al. (2001) and Brandl et al. (2016) suggested that weight gain plateaued off at 6–9 months after initiating antipsychotics. This could mean that, in some primary studies, the duration of treatment may have been too short to observe weight gain, while in other studies weight change within individuals may not be easily distinguishable in long-term antipsychotic use. Furthermore, it is common for participants who take antipsychotics to be co-prescribed other psychotropic medications, many of which are CYP2D6 substrates or inhibitors and can influence the level of antipsychotics in the plasma (Spina and De Leon, 2007; Taylor et al., 2018). Another limitation of our meta-analysis (and the literature identified more broadly) is that there were not enough studies to conduct single drug analyses and that we had to combine studies across several antipsychotics, which differ in their influence on weight and dependency of CYP2D6. Similarly, it was impossible to capture the effect of antipsychotic dose which is likely to be important on the extent of weight change, but current evidence is still limited, as summarized in Anath et al. (2003) review.

Finally, the mechanism of antipsychotic-induced weight gain is complex and involves both genetic and lifestyle factors. The mechanism is polygenic with strong genetic influence as demonstrated in a monozygotic twin study (Theisen et al., 2005). There has been extensive evidence on the role of *HTR2C* gene (De Luca et al., 2007; Wallace et al., 2011) and many genome-wide association studies have identified candidate genes for antipsychotic induced weight gain, supporting the importance of genetics in what is a complex gene-drug interaction (Müller and Kennedy, 2006; Brandl et al., 2016; Maciukiewicz et al., 2019). Considering lifestyle factors, many people with antipsychotic-induced weight gain concurrently have disturbances in other metabolic markers, including hypercholesterolemia, raised HbA1c or plasma glucose. It is believed that one mechanism is an increase in leptin plasma levels, a hormone that increases appetite and food intake (Reynolds and McGowan, 2017). Future research should account for lifestyle factors influencing weight and consider background genetic variation, for example, by using polygenic risk scores of antipsychotic-induced weight gain.

In conclusion, our systematic review of cohort studies suggested that CYP2D6 PMs taking antipsychotics have higher BMI than NMs. Of note, these four studies were designed to control variables affecting weight and BMI and included one of the largest and most comprehensively genotyped samples. However, the rest of the systematic review in cross-sectional studies, and the meta-analysis, did not find an influence of *CYP2D6* genetic variation on antipsychotic-induced weight or BMI gain. This literature is still limited by the small number of participants with genetic variants resulting in PM status and the high heterogeneity between studies. In addition, BMI and weight gain can be influenced by environmental and genetic factors, which cannot all be accounted for in the same way across the literature. Overall, our review suggested that there may be some association between *CYP2D6* gene variation and antipsychotic-induced weight gain, but current evidence is not sufficient to confirm the relationship. Therefore, prospective studies with large samples, especially of PMs, are needed to explore this relationship further. Nevertheless, *CYP2D6* remains the primary candidate gene for genetic testing to guide antipsychotic prescribing as recommended by the Dutch Pharmacogenetics Working Group (Swen et al., 2011). With further research and collaborative effort to incorporate pharmacogenetics into clinical environment, *CYP2D6* testing may prove useful to prevent or mitigate weight gain and other adverse effects in people needing antipsychotic treatment.

## Data Availability Statement

The original contributions presented in the study are included in the article/Supplementary Material, further inquiries can be directed to the corresponding author/s.

## Author Contributions

YW, IA-Z, and EB designed the study and the statistical analysis plan. YW conducted the search and statistical analysis, with support from IA-Z and EB. SA, GJ, HR, MA, RC, and SK provided the additional data. YW wrote the manuscript, with contributions from SA, MA, RC, GJ, SK, HR, AB, HI, DK, RP, BW, EZ, IA-Z, and EB. YW, SA, MA, RC, GJ, SK, HR, AB, HI, DK, RP, BW, EZ, IA-Z, and EB reviewed the manuscript and made substantial contributions to the writing and interpretation. All the authors contributed to the article and approved the submitted version.

## Conflict of Interest

The authors declare that the research was conducted in the absence of any commercial or financial relationships that could be construed as a potential conflict of interest.

## Publisher’s Note

All claims expressed in this article are solely those of the authors and do not necessarily represent those of their affiliated organizations, or those of the publisher, the editors and the reviewers. Any product that may be evaluated in this article, or claim that may be made by its manufacturer, is not guaranteed or endorsed by the publisher.
