# Supplementary material for: CYP2D6 Genetic Variation and Antipsychotic-Induced Weight Gain: A Systematic Review and Meta-Analysis
Source: Front Psychol. 2022 Feb 3;12:768748. doi: 10.3389/fpsyg.2021.768748 (PMC8850377; doi:10.3389/fpsyg.2021.768748)
Supplement: Supplementary file 1 [file Data_Sheet_1.docx]

Supplementary Material

# Supplementary Tables

**Supplementary Table 1**: **conversion from star alleles to activity scores**.

| **Functional status** | **Activity score** | **Example alleles** |
| --- | --- | --- |
| Increased function | >1 | *1xN, *2xN |
| Normal function | 1 | *1, *2, *27, *33 |
| Decreased function | 0.5  0.25 | *9, *14B, *17, *29, *41  *10 |
| No function | 0 | *3, *4, *5, *6, *7, *8, *14A, *19 |

**Supplementary Table 2**: **conversion from total activity score to metabolic status.** **N represents the duplication of a functional CYP2D6 gene and N is the number of copies of a haplotype.*

| **Metabolic status** | **Total activity score** | **Example diplotypes** |
| --- | --- | --- |
| Ultra-rapid metabolizers | > 2.25 | *1/*1N, *1/*2N |
| Normal metabolizers | 1.25  ≤≤   x  ≤≤   2.25 | *1/*1, *1/*10, *1/*41 |
| Intermediate metabolizers | 0.25  ≤≤   x  ≤≤    1 | *4/*10, *10/*10, |
| Poor metabolizers | 0 | *5/*5, *4/*4, *3/*4 |

**Supplementary Table 3**: **Table showing a list of 189 records that were fully assessed by reviewers and reasons for excluding them.**

| **Record** | **Reason for exclusion** | **PMID** | **First author** | **Year** | **Title** |
| --- | --- | --- | --- | --- | --- |
| 1 | Included in meta-analysis | 32643213 | [Sanne Maartje Kloosterboer](https://bpspubs-onlinelibrary-wiley-com.libproxy.ucl.ac.uk/action/doSearch?ContribAuthorStored=Kloosterboer%2C+Sanne+Maartje) | 2020 | Risperidone plasma concentrations are associated with side effects and effectiveness in children and adolescents with autism spectrum disorder. |
| 2 | Included in both | 32845723 | Jallaq | 2020 | CYP2D6 Phenotype Influences Aripiprazole Tolerability in Pediatric Patients with Mood Disorders. |
| 3 | Included in meta-analysis | 31346157 | Arranz | 2019 | A pharmacogenetic intervention for the improvement of the safety profile of antipsychotic treatments. |
| 4 | Included in systematic review | 29369497 | Sukasem | 2018 | Pharmacogenetics of Risperidone-Induced Insulin Resistance in Children and Adolescents with Autism Spectrum Disorder. |
| 5 | Included in systematic review | 27932669 | Akamine | 2016 | Quantification of the steady-state plasma concentrations of clozapine and N-desmethylclozapine in Japanese patients with schizophrenia using a novel HPLC method and the effects of CYPs and ABC transporters polymorphisms. |
| 6 | Included in systematic review | 27875318 | Sychev | 2016 | 1846G&gt;A polymorphism of CYP2D6 gene and extrapyramidal side effects during antipsychotic therapy among Russians and Tatars: a pilot study. |
| 7 | Included in meta-analysis | 27021090 | Ivanova | 2016 | CYP1A2 and CYP2D6 Gene Polymorphisms in Schizophrenic Patients with Neuroleptic Drug-Induced Side Effects. |
| 8 | Included in systematic review | 26937359 | Dodgen | 2016 | Risperidone-associated adverse drug reactions and CYP2D6 polymorphisms in a South African cohort. |
| 9 | Included in both | 26880915 | Dos Santos-Júnior | 2016 | Pharmacogenetics of Risperidone and Cardiovascular Risk in Children and Adolescents. |
| 10 | Included in both | 25329115 | Nussbaum | 2014 | Molecular study of weight gain related to atypical antipsychotics: clinical implications of the CYP2D6 genotype. |
| 11 | Included in both | 24589909 | Suzuki | 2014 | Effect of risperidone metabolism and P-glycoprotein gene polymorphism on QT interval in patients with schizophrenia. |
| 12 | Included in meta-analysis | 21519338 | Bigos | 2011 | Genetic variation in CYP3A43 explains racial difference in olanzapine clearance. |
| 13 | Included in systematic review | 19997080 | Correia | 2009 | Pharmacogenetics of risperidone therapy in autism: association analysis of eight candidate genes with drug efficacy and adverse drug reactions. |
| 14 | Included in meta-analysis | 12766554 | Mihara | 2003 | Effects of various CYP2D6 genotypes on the steady-state plasma concentrations of risperidone and its active metabolite, 9-hydroxyrisperidone, in Japanese patients with schizophrenia. |
| 15 | Included in both | 11901361 | Ellingrod | 2002 | CYP2D6 polymorphisms and atypical antipsychotic weight gain. |
| 16 | Included in meta-analysis | 10850389 | Mihara | 2000 | Effect of a genetic polymorphism of CYP1A2 inducibility on the steady state plasma concentrations of haloperidol and reduced haloperidol in Japanese patients with schizophrenia. |
| 17 | Included in systematic review | 33535976 | Lu | 2021 | Effect of CYP2D6 polymorphisms on plasma concentration and therapeutic effect of risperidone. |
| 18 | Included in meta-analysis | 33336447 | Ortega-Vazquez | 2020 | Alcohol intake potentiates clozapine adverse effects associated to CYP1A2*1C in patients with refractory psychosis. |
| 19 | Included in meta-analysis | 33284338 | Jurgens | 2020 | Effect of Routine Cytochrome P450 2D6 and 2C19 Genotyping on Antipsychotic Drug Persistence in Patients With Schizophrenia: A Randomized Clinical Trial. |
| 20 | Included in meta-analysis | 30604050 | Kiss | 2019 | Phenoconversion of CYP2D6 by inhibitors modifies aripiprazole exposure. |
| 21 | Included in systematic review | 16633140 | Lane | 2006 | Risperidone-related weight gain: genetic and nongenetic predictors |
| 22 | Included in systematic review |  | Vanwong | 2014 | Pharmacogenetics and clinical risk factors for risperidone-related weight gain in Thai Autistic Spectrum disorder patients. |
| 23 | Included in meta-analysis | 23851570 | Roke | 2013 | The effect of the Taq1A variant in the dopamine D(2) receptor gene and common CYP2D6 alleles on prolactin levels in risperidone-treated boys. |
| 24 | No weight or BMI measurements | 30864366 | Filipce | 2019 | Evaluation of Correlation Between the Pharmacogenetic Profiles of Risperidone Treated Psychiatry Patients with Plasma and Urine Concentration of Risperidone and its Active Moiety 9-OH Risperidone Determined with Optimized Bioanalytical LC Method. |
| 25 | No weight or BMI measurements | 29699889 | Walden | 2018 | Genetic testing for CYP2D6 and CYP2C19 suggests improved outcome for antidepressant and antipsychotic medication |
| 26 | No weight or BMI measurements | 27378571 | Lloret-Linares | 2016 | Screening for genotypic and phenotypic variations in CYP450 activity in patients with therapeutic problems in a psychiatric setting, a retrospective study. |
| 27 | No weight or BMI measurements | 29783455 | Subuh Surja | 2008 | Pharmacogenetic testing of CYP2D6 in patients with aripiprazole-related extrapyramidal symptoms: a case-control study. |
| 28 | No weight or BMI measurements | 16478753 | Plesnicar | 2006 | The influence of the CYP2D6 polymorphism on psychopathological and extrapyramidal symptoms in the patients on long-term antipsychotic treatment. |
| 29 | No weight or BMI measurements | 12960748 | Inada | 2003 | Cytochrome P450 II D6 gene polymorphisms and the neuroleptic-induced extrapyramidal symptoms in Japanese schizophrenic patients. |
| 30 | No weight or BMI measurements | 9564684 | Jönsson | 1998 | Lack of association between debrisoquine 4-hydroxylase (CYP2D6) gene polymorphisms and schizophrenia. |
| 31 | No weight or BMI measurements | 25254417 | Bakken | 2015 | Impact of genetic variability in CYP2D6, CYP3A5, and ABCB1 on serum concentrations of quetiapine and N-desalkylquetiapine in psychiatric patients |
| 32 | No weight or BMI measurements | 20563569 | Jovanovic | 2010 | The role of CYP2D6 and ABCB1 pharmacogenetics in drug-naïve patients with first-episode schizophrenia treated with risperidone. |
| 33 | Same dataset used. | 26682995 | dos Santos Júnior | 2015 | Hyperprolactinemia in Children and Adolescents with Use of Risperidone: Clinical and Molecular Genetics Aspects. |
| 34 | Authors contacted but unable to obtain enough data | 28719598 | Rafaniello | 2017 | The predictive value of ABCB1, ABCG2, CYP3A4/5 and CYP2D6 polymorphisms for risperidone and aripiprazole plasma concentrations and the occurrence of adverse drug reactions. |
| 35 | Authors contacted but unable to obtain enough data | 28340122 | Tóth | 2017 | Potential Role of Patients' CYP3A-Status in Clozapine Pharmacokinetics. |
| 36 | Authors contacted but unable to obtain enough data | 26488675 | Hwang | 2015 | Fast Versus Slow Strategy of Switching Patients With Schizophrenia to Aripiprazole From Other Antipsychotics. |
| 37 | Authors contacted but unable to obtain enough data | 26418700 | Mannheimer | 2015 | Risperidone and Venlafaxine Metabolic Ratios Strongly Predict a CYP2D6 Poor Metabolizing Genotype. |
| 38 | Authors contacted but unable to obtain enough data | 24828442 | Youngster | 2014 | CYP2D6 genotyping in paediatric patients with autism treated with risperidone: a preliminary cohort study. |
| 39 | Authors contacted but unable to obtain enough data | 23868656 | Gomeni | 2013 | A model-based approach to characterize the population p |
| 40 | Authors contacted but unable to obtain enough data | 22929407 | Sherwin | 2012 | Population pharmacokinetic modeling of risperidone and 9-hydroxyrisperidone to estimate CYP2D6 subpopulations in children and adolescents. |
| 41 | Authors contacted but unable to obtain enough data | 22722500 | Lee | 2012 | Association study of 27 annotated genes for clozapine pharmacogenetics: validation of preexisting studies and identification of a new candidate gene, ABCB1, for treatment response. |
| 42 | Authors contacted but unable to obtain enough data | 22198443 | Jürgens | 2011 | Does the medication pattern reflect the CYP2D6 genotype in patients with diagnoses within the schizophrenic spectrum? |
| 43 | Authors contacted but unable to obtain enough data | 19954080 | Gonzalez-Tejera | 2009 | CYP2D6 genotypes in Puerto Rican psychiatry patients with intolerance of antidepressants and antipsychotics. |
| 44 | Authors contacted but unable to obtain enough data | 19521114 | Kobylecki | 2009 | CYP2D6 genotype predicts antipsychotic side effects in schizophrenia inpatients: a retrospective matched case-control study. |
| 45 | Authors contacted but unable to obtain enough data | 18346175 | Crescenti | 2008 | Cyp2d6*3, *4, *5 and *6 polymorphisms and antipsychotic-induced extrapyramidal side-effects in patients receiving antipsychotic therapy. |
| 46 | Authors contacted but unable to obtain enough data | 17701031 | Kato | 2007 | Effects of CYP2D6 polymorphisms on neuroleptic malignant syndrome. |
| 47 | Authors contacted but unable to obtain enough data | 17136449 | Vermeulen | 2006 | Population pharmacokinetics of risperidone and 9-hydroxyrisperidone in patients with acute episodes associated with bipolar I disorder. |
| 48 | Authors contacted but unable to obtain enough data | 16160620 | de Leon | 2005 | Polymorphic variations in GSTM1, GSTT1, PgP, CYP2D6, CYP3A5, and dopamine D2 and D3 receptors and their association with tardive dyskinesia in severe mental illness. |
| 49 | Authors contacted but unable to obtain enough data | 15820320 | Tiwari | 2005 | Genetic susceptibility to tardive dyskinesia in chronic schizophrenia subjects: III. Lack of association of CYP3A4 and CYP2D6 gene polymorphisms. |
| 50 | Authors contacted but unable to obtain enough data | 15669884 | de Leon | 2005 | The CYP2D6 poor metabolizer phenotype may be associated with risperidone adverse drug reactions and discontinuation. |
| 51 | Authors contacted but unable to obtain enough data | 15538130 | de Leon | 2004 | Haloperidol half-life after chronic dosing. |
| 52 | Authors contacted but unable to obtain enough data | 15118351 | Liou | 2004 | Cytochrome P-450 2D6*10 C188T polymorphism is associated with antipsychotic-induced persistent tardive dyskinesia in Chinese schizophrenic patients. |
| 53 | Authors contacted but unable to obtain enough data | 14499311 | Ohara | 2003 | Effects of smoking and cytochrome P450 2D6*10 allele on the plasma haloperidol concentration/dose ratio. |
| 54 | Authors contacted but unable to obtain enough data | 12734765 | Lohmann | 2003 | CYP2D6 polymorphism and tardive dyskinesia in schizophrenic patients. |
| 55 | Authors contacted but unable to obtain enough data | 12691769 | Ohara | 2003 | Effects of age and the CYP2D6*10 allele on the plasma haloperidol concentration/dose ratio. |
| 56 | Authors contacted but unable to obtain enough data | 12629505 | Nikoloff | 2003 | Association between CYP2D6 genotype and tardive dyskinesia in Korean schizophrenics. |
| 57 | Authors contacted but unable to obtain enough data | 12432967 | Ellingrod | 2002 | Abnormal movements and tardive dyskinesia in smokers and nonsmokers with schizophrenia genotyped for cytochrome P450 2D6. |
| 58 | Authors contacted but unable to obtain enough data | 12386646 | Brockmöller | 2002 | The impact of the CYP2D6 polymorphism on haloperidol pharmacokinetics and on the outcome of haloperidol treatment. |
| 59 | Authors contacted but unable to obtain enough data | 12107620 | Jaanson | 2002 | Maintenance therapy with zuclopenthixol decanoate: associations between plasma concentrations, neurological side effects and CYP2D6 genotype. |
| 60 | Authors contacted but unable to obtain enough data | 11778144 | Lam | 2002 | Cytochrome P450 2D6 genotyping and association with tardive dyskinesia in Chinese schizophrenic patients. |
| 61 | Authors contacted but unable to obtain enough data | 10942177 | Shimoda | 2000 | CYP2D6*10 alleles are not the determinant of the plasma haloperidol concentrations in Asian patients. |
| 62 | Authors contacted but unable to obtain enough data | 10909122 | Ellingrod | 2000 | Association between cytochrome P4502D6 (CYP2D6) genotype, antipsychotic exposure, and abnormal involuntary movement scale (AIMS) score. |
| 63 | Authors contacted but unable to obtain enough data | 9713906 | Ohmori | 1998 | Tardive dyskinesia and debrisoquine 4-hydroxylase (CYP2D6) genotype in Japanese schizophrenics. |
| 64 | Authors contacted but unable to obtain enough data | 9713905 | Kapitany | 1998 | Genetic polymorphisms for drug metabolism (CYP2D6) and tardive dyskinesia in schizophrenia. |
| 65 | Authors contacted but unable to obtain enough data | 9564684 | Jönsson | 1998 | Lack of association between debrisoquine 4-hydroxylase (CYP2D6) gene polymorphisms and schizophrenia. |
| 66 | Authors contacted but unable to obtain enough data | 9539256 | Suzuki | 1998 | Effects of various factors including the CYP2D6 genotype and coadministration of flunitrazepam on the steady-state plasma concentrations of bromperidol and its reduced metabolite. |
| 67 | Authors contacted but unable to obtain enough data | 9323326 | Kawanishi | 1997 | Neuroleptic malignant syndrome and hydroxylase gene mutations: no association with CYP2D6A or CYP2D6B. |
| 68 | Authors contacted but unable to obtain enough data | 9201806 | Andreassen | 1997 | Non-functional CYP2D6 alleles and risk for neuroleptic-induced movement disorders in schizophrenic patients. |
| 69 | Authors contacted but unable to obtain enough data | 9068770 | Armstrong | 1997 | Antipsychotic drug-induced movement disorders in schizophrenics in relation to CYP2D6 genotype. |
| 70 | Authors contacted but unable to obtain enough data | 7635999 | Arthur | 1995 | Polymorphic drug metabolism in schizophrenic patients w |
| 71 | Authors contacted but unable to obtain enough data | 25266547 | Turncliff | 2014 | Relative bioavailability and safety of aripiprazole lauroxil, a novel once-monthly, long-acting injectable atypical antipsychotic, following deltoid and gluteal administration in adult subjects with schizophrenia |
| 72 | Authors contacted but unable to obtain enough data | 11147929 | Dettling | 2000 | Clozapine-induced agranulocytosis and hereditary polymorphisms of clozapine metabolizing enzymes: no association with myeloperoxidase and cytochrome P4502D6 |
| 73 | Authors contacted but unable to obtain enough data | 33361064 | Tsermpini | 2020 | Clinical implementation of preemptive pharmacogenomics in psychiatry: Τhe &quot;PREPARE&quot; study. |
| 74 | Authors contacted but unable to obtain enough data | 17667795 | Panagiotidis | 2007 | Depot haloperidol treatment in outpatients with schizophrenia on monotherapy: impact of CYP2D6 polymorphism on pharmacokinetics and treatment outcome. |
| 75 | Authors contacted but unable to obtain enough data | 26944100 | Vanwong | 2016 | Detection of CYP2D6 polymorphism using Luminex xTAG technology in autism spectrum disorder: CYP2D6 activity score and its association with risperidone levels. |
| 76 | Authors contacted but unable to obtain enough data | 26780783 | Vanwong | 2017 | Impact of CYP2D6 polymorphism on steady-state plasma levels of risperidone and 9-hydroxyrisperidone in Thai children and adolescents with autism spectrum disorder. |
| 77 | Authors contacted but unable to obtain enough data |  | Sukasem | 2016 | Impact of pharmacogenetic markers of CYP2D6 and DRD2 on prolactin response in risperidone-treated Thai children and adolescents with autism spectrum disorders. |
| 78 | Authors contacted but unable to obtain enough data | 17224713 | Troost | 2007 | Prolactin release in children treated with risperidone: impact and role of CYP2D6 metabolism. |
| 79 | Authors contacted but unable to obtain enough data | 24026091 | Amoguera | 2013 | CYP2D6 poor metabolizer status might be associated with better response to risperidone treatment. |
| 80 | Authors contacted but unable to obtain enough data | 26129906 | Vandenberghe | 2015 | Genetics-based population pharmacokinetics and pharmacodynamics of risperidone in a psychiatric cohort. |
| 81 | Authors contacted but unable to obtain enough data | 11214775 | Scordo | 2000 | CYP2D6 genotype and antipsychotic-induced extrapyramidal side effects in schizophrenic patients. |
| 82 | Authors contacted but unable to obtain enough data | 15729081 | Kakihara | 2005 | Prediction of response to risperidone treatment with respect to plasma concentrations of risperidone, catecholamine metabolites, and polymorphism of cytochrome P450 2D6 |
| 83 | Authors contacted but unable to obtain enough data | 24595968 | Koola | 2014 | Association of tardive dyskinesia with variation in CYP2D6: is there a role for active metabolites? |
| 84 | Authors contacted but unable to obtain enough data | 19475583 | Tsai | 2010 | A candidate gene study of tardive dyskinesia in the CATIE schizophrenia trial. |
| 85 | Did not genotype CYP2D6 | 32767297 | Schoretsanitis | 2020 | Lack of Smoking Effects on Pharmacokinetics of Oral Paliperidone-analysis of a Naturalistic Therapeutic Drug Monitoring Sample. |
| 86 | Did not genotype CYP2D7 | 32519194 | Hefner | 2020 | Prevalence and sort of pharmacokinetic drug-drug interactions in hospitalized psychiatric patients. |
| 87 | Did not genotype CYP2D8 | 31919994 | Sun | 2020 | Physiologically-Based Pharmacokinetic Modeling for Predicting Drug Interactions of a Combination of Olanzapine and Samidorphan. |
| 88 | Did not genotype CYP2D9 | 29343979 | Zastrozhin | 2018 | The influence of CYP3A5 polymorphisms on haloperidol treatment in patients with alcohol addiction. |
| 89 | Did not genotype CYP2D10 | 28664816 | Piatkov | 2017 | CYP2C19*17 protects against metabolic complications of clozapine treatment. |
| 90 | Did not genotype CYP2D11 | 28160505 | Paulzen | 2017 | Cytochrome P450-mediated interaction between perazine and risperidone: implications for antipsychotic polypharmacy. |
| 91 | Did not genotype CYP2D12 | 27639091 | Tóth | 2016 | Optimization of Clonazepam Therapy Adjusted to Patient's CYP3A Status and NAT2 Genotype. |
| 92 | Did not genotype CYP2D13 | 27681143 | Vasudev | 2016 | Genetic Determinants of Clozapine-Induced Metabolic Side Effects. |
| 93 | Did not genotype CYP2D14 | 27448523 | Paulzen | 2016 | Body mass index (BMI) but not body weight is associated with changes in the metabolism of risperidone; A pharmacokinetics-based hypothesis. |
| 94 | Did not genotype CYP2D15 | 26137357 | Olsson | 2015 | Genetic and Clinical Factors Affecting Plasma Clozapine Concentration. |
| 95 | Did not genotype CYP2D16 | 25602162 | Ivanova | 2015 | Cytochrome P450 1A2 co-determines neuroleptic load and may diminish tardive dyskinesia by increased inducibility. |
| 96 | Did not genotype CYP2D17 | 25090458 | Czerwensky | 2014 | CYP1A2*1D and *1F polymorphisms have a significant impact on olanzapine serum concentrations. |
| 97 | Did not genotype CYP2D18 | 24074484 | Kamijima | 2013 | Aripiprazole augmentation to antidepressant therapy in Japanese patients with major depressive disorder: a randomized, double-blind, placebo-controlled study (ADMIRE study). |
| 98 | Did not genotype CYP2D19 | 22901441 | Ferrari | 2012 | Association between CYP1A2 polymorphisms and clozapine-induced adverse reactions in patients with schizophrenia. |
| 99 | Did not genotype CYP2D20 | 21486167 | Calarge | 2011 | Predictors of risperidone and 9-hydroxyrisperidone serum concentration in children and adolescents. |
| 100 | Did not genotype CYP2D21 | 20143052 | Ghotbi | 2010 | Carriers of the UGT1A4 142T&gt;G gene variant are predisp |
| 101 | Did not genotype CYP2D22 | 19636338 | Laika | 2009 | Pharmacogenetics and olanzapine treatment: CYP1A2*1F and serotonergic polymorphisms influence therapeutic outcome. |
| 102 | Did not genotype CYP2D23 | 18666802 | Darby | 2008 | Long-term therapeutic drug monitoring of risperidone and olanzapine identifies altered steady-state pharmacokinetics: a clinical, two-group, naturalistic study. |
| 103 | Did not genotype CYP2D24 | 17688403 | Boke | 2007 | Association of serotonin 2A receptor and lack of association of CYP1A2 gene polymorphism with tardive dyskinesia in a Turkish population. |
| 104 | Did not genotype CYP2D25 | 16490169 | Fu | 2006 | Association of CYP2D6 and CYP1A2 gene polymorphism with tardive dyskinesia in Chinese schizophrenic patients. |
| 105 | Did not genotype CYP2D26 | 15982995 | Aichhorn | 2005 | Influence of age and gender on risperidone plasma concentrations. |
| 106 | Did not genotype CYP2D27 | 15949157 | Kootstra-Ros | 2005 | The cytochrome P450 CYP1A2 genetic polymorphisms *1F and *1D do not affect clozapine clearance in a group of schizophrenic patients. |
| 107 | Did not genotype CYP2D28 | 15505641 | Tiwari | 2004 | Genetic susceptibility to tardive dyskinesia in chronic schizophrenia subjects: I. Association of CYP1A2 gene polymorphism. |
| 108 | Did not genotype CYP2D29 | 15289794 | Faber | 2004 | Time response of cytochrome P450 1A2 activity on cessation of heavy smoking. |
| 109 | Did not genotype CYP2D30 | 14659489 | Doude van Troostwijk | 2003 | CYP1A2 activity is an important determinant of clozapine dosage in schizophrenic patients. |
| 110 | Did not genotype CYP2D31 | 12188102 | Dailly | 2002 | Evidence from a population pharmacokinetics analysis for a major effect of CYP1A2 activity on inter- and intraindividual variations of clozapine clearance. |
| 111 | Did not genotype CYP2D32 | 11839369 | Segman | 2002 | Interactive effect of cytochrome P450 17alpha-hydroxylase and dopamine D3 receptor gene polymorphisms on abnormal involuntary movements in chronic schizophrenia. |
| 112 | Did not genotype CYP2D33 | 11817502 | Shimoda | 2002 | Lack of impact of CYP1A2 genetic polymorphism (C/A polymorphism at position 734 in intron 1 and G/A polymorphism at position -2964 in the 5'-flanking region of CYP1A2) on the plasma concentration of haloperidol in smoking male Japanese with schizophrenia. |
| 113 | Did not genotype CYP2D34 | 11496364 | Schulze | 2001 | Lack of association between a functional polymorphism of the cytochrome P450 1A2 (CYP1A2) gene and tardive dyskinesia in schizophrenia. |
| 114 | Did not genotype CYP2D35 | 11476124 | Ozdemir | 2001 | CYP1A2 activity as measured by a caffeine test predicts clozapine and active metabolite steady-state concentrationin patients with schizophrenia. |
| 115 | Did not genotype CYP2D36 | 11179771 | Ozdemir | 2001 | Pharmacogenetic assessment of antipsychotic-induced movement disorders: contribution of the dopamine D3 receptor and cytochrome P450 1A2 genes. |
| 116 | Did not genotype CYP2D37 | 10942191 | Spina | 2000 | Plasma concentrations of risperidone and 9-hydroxyrisperidone: effect of comedication with carbamazepine or valproate. |
| 117 | Did not genotype CYP2D38 | 10889552 | Basile | 2000 | A functional polymorphism of the cytochrome P450 1A2 (CYP1A2) gene: association with tardive dyskinesia in schizophrenia. |
| 118 | Did not genotype CYP2D39 | 10365650 | Facciolà | 1999 | Small effects of valproic acid on the plasma concentrations of clozapine and its major metabolites in patients with schizophrenic or affective disorders. |
| 119 | Did not genotype CYP2D40 | 10051063 | Balant-Gorgia | 1999 | Therapeutic drug monitoring of risperidone using a new, rapid HPLC method: reappraisal of interindividual variability factors. |
| 120 | Did not genotype CYP2D41 | 9853978 | Facciolà | 1998 | Inducing effect of phenobarbital on clozapine metabolism in patients with chronic schizophrenia. |
| 121 | Did not genotype CYP2D42 | N/A (CENTRAAL) | Citrome | 2007 | Pharmacokinetics of aripiprazole and concomitant carbamazepine |
| 122 | Did not genotype CYP2D43 | N/A (PsychInfo) | de Brito | 2015 | The CYP1A2 -163CNA polymorphism is associated with super-refractory schizophrenia. [References]. |
| 123 | Did not genotype CYP2D44 | N/A (PsychInfo) | Bolla | 2011 | Are CYP1A2*1F and *1C associated with clozapine tolerability? A preliminary investigation. |
| 124 | Did not genotype CYP2D45 | N/A (PsychInfo) | Ruan | 2019 | Exploring the prevalence of clozapine phenotypic poor metabolizers in 4 Asian samples: They ranged between 2% and 13%. [References]. |
| 125 | Did not genotype CYP2D46 | 21486167 | Calarge | 2011 | Predictors of risperidone and 9-hydroxyrisperidone serum concentration in children and adolescents. |
| 126 | Did not genotype CYP2D47 | 17688403 | Boke | 2007 | Association of serotonin 2A receptor and lack of association of CYP1A2 gene polymorphism with tardive dyskinesia in a Turkish population. |
| 127 | Did not genotype CYP2D48 | N/A | Schulze | 2001 | Lack of association between a functional polymorphism of the cytochrome P450 1A2 (CYP1A2) gene and tardive dyskinesia in schizophrenia |
| 128 | Did not genotype CYP2D49 | 33731885 | Ammar | 2021 | Clinical and genetic influencing factors on clozapine pharmacokinetics in Tunisian schizophrenic patients. |
| 129 | Did not genotype CYP2D50 | 33291155 | Fekete | 2020 | Dose-Corrected Serum Concentrations and Metabolite to Parent Compound Ratios of Venlafaxine and Risperidone from Childhood to Old Age. |
| 130 | Did not genotype CYP2D51 | 33277605 | Menus | 2020 | Association of clozapine-related metabolic disturbances with CYP3A4 expression in patients with schizophrenia. |
| 131 | Did not genotype CYP2D52 | 32119764 | de Leon | 2020 | Using therapeutic drug monitoring to personalize clozapine dosing in Asians. |
| 132 | Ineligible population | 30451558 | White | 2018 | Pharmacogenomics and Psychiatric Nursing. |
| 133 | Ineligible population | 32991788 | Koller | 2020 | Safety and cardiovascular effects of multiple-dose administration of aripiprazole and olanzapine in a randomised clinical trial. |
| 134 | Ineligible population | 30888624 | Sun | 2019 | A Phase I Open-Label Study to Evaluate the Effects of Rifampin on the Pharmacokinetics of Olanzapine and Samidorphan Administered in Combination in Healthy Human Subjects. |
| 135 | Ineligible population | 31359271 | Kneller | 2019 | Physiologically Based Pharmacokinetic Modelling to Describe the Pharmacokinetics of Risperidone and 9-Hydroxyrisperidone According to Cytochrome P450 2D6 Phenotypes. |
| 136 | Ineligible population | 29851709 | Greenblatt | 2018 | Sustained Impairment of Lurasidone Clearance After Discontinuation of Posaconazole: Impact of Obesity, and Implications for Patient Safety. |
| 137 | Ineligible population | 29325225 | Belmonte | 2018 | Influence of CYP2D6, CYP3A4, CYP3A5 and ABCB1 Polymorphisms on Pharmacokinetics and Safety of Aripiprazole in Healthy Volunteers. |
| 138 | Ineligible population | 28787271 | Sychev | 2017 | Genotyping and phenotyping of CYP2D6 and CYP3A isoenzymes in patients with alcohol use disorder: correlation with haloperidol plasma concentration. |
| 139 | Ineligible population | 27738374 | Taskin | 2016 | Investigation of CYP2D6 Gene Polymorphisms in Turkish Population. |
| 140 | Ineligible population | 26902506 | Jeon | 2016 | Population pharmacokinetics of aripiprazole in healthy Korean subjects. |
| 141 | Ineligible population | 25999696 | Bagheri | 2015 | Prevalence of the CYP2D6*10 (C100T), *4 (G1846A), and *14 (G1758A) alleles among Iranians of different ethnicities. |
| 142 | Ineligible population | 25025989 | Cabaleiro | 2014 | Pharmacogenetics of quetiapine in healthy volunteers: association with pharmacokinetics, pharmacodynamics, and adverse effects. |
| 143 | Ineligible population | 21518375 | Mahatthanatrakul | 2011 | Effect of cytochrome P450 3A4 inhibitor ketoconazole on risperidone pharmacokinetics in healthy volunteers. |
| 144 | Ineligible population | 20814331 | Novalbos | 2010 | Effects of CYP2D6 genotype on the pharmacokinetics, pharmacodynamics, and safety of risperidone in healthy volunteers. |
| 145 | Ineligible population | 17965519 | Kubo | 2007 | Pharmacokinetics of aripiprazole, a new antipsychotic, following oral dosing in healthy adult Japanese volunteers: influence of CYP2D6 polymorphism. |
| 146 | Ineligible population | 16915578 | Lee | 2006 | Pharmacokinetic parameters of bromperidol in Korean subjects. |
| 147 | Ineligible population | 16633141 | Park | 2006 | Combined effects of itraconazole and CYP2D6*10 genetic polymorphism on the pharmacokinetics and pharmacodynamics of haloperidol in healthy subjects. |
| 148 | Ineligible population | 16633151 | Hedenmalm | 2006 | Risk factors for extrapyramidal symptoms during treatment with selective serotonin reuptake inhibitors, including cytochrome P-450 enzyme, and serotonin and dopamine transporter and receptor polymorphisms. |
| 149 | Ineligible population | 16154484 | Alderman | 2005 | Coadministration of sertraline with cisapride or pimozide: an open-label, nonrandomized examination of pharmacokinetics and corrected QT intervals in healthy adult volunteers. |
| 150 | Ineligible population | 15349706 | Zackrisson | 2004 | Fatal intoxication cases: cytochrome P450 2D6 and 2C19 genotype distributions. |
| 151 | Ineligible population | 9951426 | Desta | 1999 | Effect of clarithromycin on the pharmacokinetics and pharmacodynamics of pimozide in healthy poor and extensive metabolizers of cytochrome P450 2D6 (CYP2D6). |
| 152 | Ineligible population | 9395157 | Spigset | 1997 | Seizures and myoclonus associated with antidepressant treatment: assessment of potential risk factors, including CYP2D6 and CYP2C19 polymorphisms, and treatment with CYP2D6 inhibitors. |
| 153 | Ineligible population | 9218930 | Wong | 1997 | Pharmacokinetics of sertindole and dehydrosertindole in volunteers with normal or impaired renal function. |
| 154 | Ineligible population | 24088126 | Gassa | 2013 | Relationship between CYP2D6 genotype and haloperidol pharmacokinetics and extrapyramidal symptoms in healthy volunteers |
| 155 | Ineligible population | 12746736 | Desai | 2003 | Pharmacokinetics and QT interval pharmacodynamics of oral haloperidol in poor and extensive metabolizers of CYP2D6 |
| 156 | Ineligible population | N/A (CENTRAAL) | Khorana | 2011 | Comparative pharmacokinetics and bioequivalence of two tablet formulations of 2 mg risperidone in healthy Thai male volunteers |
| 157 | Ineligible population | 8963481 | Bagli | 1995 | Bioequivalence and absolute bioavailability of oblong and coated levomepromazine tablets in CYP2D6 phenotyped subjects |
| 158 | Ineligible population | N/A (CENTRAAL) | NCT01284959, | 2011 | Different Safety Profile of Risperidone and Paliperidone Extended-release |
| 159 | Ineligible population | 23559402 | Cabaleiro | 2013 | Polymorphisms influencing olanzapine metabolism and adverse effects in healthy subjects |
| 160 | Ineligible population | 24329187 | Gassa | 2014 | Effect of CYP2D6 on risperidone pharmacokinetics and extrapyramidal symptoms in healthy volunteers: results from a pharmacogenetic clinical trial |
| 161 | Ineligible population | N/A (CENTRAAL) | Yang | 2011 | Abiraterone acetate: in metastatic castration-resistant prostate cancer |
| 162 | Ineligible population | N/A (Embase) | Belmonte | 2018 | Influence of CYP2D6, CYP3A4, CYP3A5 and ABCB1 Polymorphisms on Pharmacokinetics and Safety of Aripiprazole in Healthy Volunteers. |
| 163 | Ineligible population | 32623931 | Bhutani | 2020 | Impact of quercetin on pharmacokinetics of quetiapine: insights from in-vivo studies in wistar rats |
| 164 | Ineligible population | 26024250 | Ford | 2015 | Computational predictions of the site of metabolism of cytochrome P450 2D6 substrates: comparative analysis, molecular docking, bioactivation and toxicological implications. |
| 165 | Ineligible population | 24739264 | Jia | 2014 | Aggravation of clozapine-induced hepatotoxicity by glycyrrhetinic acid in rats. |
| 166 | Ineligible population | 24644270 | Cheng | 2014 | Machine learning-based prediction of drug-drug interactions by integrating drug phenotypic, therapeutic, chemical, and genomic properties. |
| 167 | Ineligible population | 24524664 | Quinones | 2014 | Perception of the usefulness of drug/gene pairs and barriers for pharmacogenomics in Latin America. |
| 168 | Ineligible population | 24185126 | Dragovic | 2013 | Effect of human glutathione S-transferase hGSTP1-1 polymorphism on the detoxification of reactive metabolites of clozapine, diclofenac and acetaminophen. |
| 169 | Ineligible population | 21890734 | Rea | 2011 | Role of residue 87 in the activity and regioselectivity of clozapine metabolism by drug-metabolizing CYP102A1 M11H: application for structural characterization of clozapine GSH conjugates. |
| 170 | Ineligible population | 20849150 | Dragovic | 2010 | Role of human glutathione S-transferases in the inactivation of reactive metabolites of clozapine. |
| 171 | Ineligible population | 18332080 | Bauman | 2008 | Comparison of the bioactivation potential of the antidepressant and hepatotoxin nefazodone with aripiprazole, a structural analog and marketed drug. |
| 172 | Ineligible population | 17329294 | Davies | 2007 | PRN prescribing in psychiatric inpatients: potential for pharmacokinetic drug interactions. |
| 173 | Ineligible population | 16538186 | Casley | 2006 | Assay for the simultaneous detection of the *1C and *1F alleles of the CYP1A2 gene by real-time polymerase chain reaction and melting curve analysis. |
| 174 | Ineligible population | 15807986 | Jang | 2005 | Effects of green tea extract administration on the pharmacokinetics of clozapine in rats. |
| 175 | Ineligible population | 15039292 | Yamazaki | 2004 | Comparison of prediction methods for in vivo clearance of (S,S)-3-[3-(methylsulfonyl)phenyl]-1-propylpiperidine hydrochloride, a dopamine D2 receptor antagonist, in humans. |
| 176 | Ineligible population | 11198052 | Aitchison | 2001 | Clozapine pharmacokinetics and pharmacodynamics studied with Cyp1A2-null mice. |
| 177 | Ineligible population | 10693156 | Prince | 2000 | Putamen mitochondrial energy metabolism is highly correlated to emotional and intellectual impairment in schizophrenics. |
| 178 | Ineligible population | 10520731 | Bun | 1999 | Interspecies variability and drug interactions of clozapine metabolism by microsomes. |
| 179 | Ineligible population | 20599499 | Locatelli | 2010 | A population pharmacokinetic evaluation of the influence of CYP2D6 genotype on risperidone metabolism in patients with acute episode of schizophrenia. |
| 180 | Ineligible population | 33378980 | Zabiaur | 2021 | Impact of polymorphisms in transporter and metabolizing enzyme genes on olanzapine pharmacokinetics and safety in healthy volunteers. |
| 181 | Ineligible population | 33278020 | Koller | 2020 | Metabolic Effects of Aripiprazole and Olanzapine Multiple-Dose Treatment in a Randomised Crossover Clinical Trial in Healthy Volunteers: Association with Pharmacogenetics. |
| 182 | Not in English | 17599129 | Hendset | 2007 | [Why measure drug metabolites?]. |
| 183 | Not in English | 17013776 | Kirchheiner | 2006 | [State of the art of pharmacogenetic diagnostics in drug therapy]. |
| 184 | Not in English | 15164612 | Shimoda | 2004 | [Effect of smoking on pharmacokinetics of antipsychotics]. |
| 185 | Not in English | 12876958 | Yoshimasu | 2003 | [Antipsychotics: side effects and drug interaction]. |
| 186 | Not in English | 11704898 | Rao | 2001 | [Olanzapine: pharmacology, pharmacokinetics and therapeutic drug monitoring]. |
| 187 | Not in English | N/A (Embase) | Anzenbacher | 2012 | Personalized medicine - Anticipation of possibilities. |
| 188 | Not in English | N/A (Embase) | Farre | 2012 | Pharmacogenetics of antipsychotic adverse effects in patients with schizophrenia. [Spanish] |
| 189 | Not in English | N/A (Embase) | Mohr | 2020 | Lurasidone: A profile of the second-generation antipsychotic. [Czech] |
